# Genetic detection of two novel *LRP5* pathogenic variants in patients with familial exudative vitreoretinopathy

**DOI:** 10.1186/s12886-023-03243-2

**Published:** 2023-11-29

**Authors:** Jiayu Li, Chanjuan Wang, Shaochi Zhang, Bo Cai, Bo Pan, Caihong Sun, Xiaolong Qi, Chunmei Ma, Wei Fang, Kangxin Jin, Xiaojun Bi, Zibing Jin, Wenjuan Zhuang

**Affiliations:** 1 Third Clinical Medical College of Ningxia Medical University, Shengli Street, Yinchuan, 750004 Ningxia China; 2 Ningxia Eye Hospital, People’s Hospital of Ningxia Hui Autonomous Region, Huanghe Road, Yinchuan, 750011 Ningxia China; 3 Beijing Institute of Ophthalmology, Beijing Tongren Eye Center, Beijing Tongren Hospital, Capital Medical University, Beijing, 100005 China

**Keywords:** Familial Exudative Vitreoretinophay (FEVR), Genetic variant, *LRP5*, Molecular dynamics simulation, Molecular docking, DKK1

## Abstract

**Background:**

Familial exudative vitreoretinopathy (FEVR) is a genetic eye disorder that leads to abnormal development of retinal blood vessels, resulting in vision impairment. This study aims to identify pathogenic variants by targeted exome sequencing in 9 independent pedigrees with FEVR and characterize the novel pathogenic variants by molecular dynamics simulation.

**Methods:**

Clinical data were collected from 9 families with FEVR. The causative genes were screened by targeted next-generation sequencing (TGS) and verified by Sanger sequencing. In silico analyses (SIFT, Polyphen2, Revel, MutationTaster, and GERP + +) were carried out to evaluate the pathogenicity of the variants. Molecular dynamics was simulated to predict protein conformation and flexibility transformation alterations on pathogenesis. Furthermore, molecular docking techniques were employed to explore the interactions and binding properties between LRP5 and DKK1 proteins relevant to the disease.

**Results:**

A 44% overall detection rate was achieved with four variants including c.4289delC: p.Pro1431Argfs*8, c.2073G > T: p.Trp691Cys, c.1801G > A: p.Gly601Arg in *LRP5* and c.633 T > A: p.Tyr211* in *TSPAN12* in 4 unrelated probands. Based on in silico analysis and ACMG standard, two of them, c.4289delC: p.Pro1431Argfs*8 and c.2073G > T: p.Trp691Cys of *LRP5* were identified as novel pathogenic variants. Based on computational predictions using molecular dynamics simulations and molecular docking, there are indications that these two variants might lead to alterations in the secondary structure and spatial conformation of the protein, potentially impacting its rigidity and flexibility. Furthermore, these pathogenic variants are speculated to potentially influence hydrogen bonding interactions and could result in an increased binding affinity with the DKK1 protein.

**Conclusions:**

Two novel genetic variants of the *LRP5* gene were identified, expanding the range of mutations associated with FEVR. Through molecular dynamics simulations and molecular docking, the potential impact of these variants on protein structure and their interactions with the DKK1 protein has been explored. These findings provide further support for the involvement of these variants in the pathogenesis of the disease.

**Supplementary Information:**

The online version contains supplementary material available at 10.1186/s12886-023-03243-2.

## Introduction

Familial exudative vitreoretinopathy (FEVR) is a highly heterogeneous and monogenic genetic disorder that affects the development of retinal blood vessels. FEVR presents with diverse signs such as peripheral retina perfusion deficiency, neovascularization, vitreoretinal traction, macular displacement, retinal folds, and retinal detachment (RD). Interestingly, family members carrying the same pathogenic variant may display varying clinical features, including complete blindness, or remain asymptomatic. Nonetheless, this disease has not exhibited gender differences [1–3]. However, due to the incomplete penetrance of the disease and the similarities in fundus manifestations with other retinal diseases, such as high myopia or retinopathy of prematurity (ROP), the disorder can be prone to underdiagnosis or misdiagnosis [4–6]. Therefore, a combination of fundus imaging and genetic screening is crucial for the diagnosis and genetic counseling of FEVR.

FEVR exhibits various inheritance patterns, including autosomal dominant (AD), autosomal recessive (AR), and X-linked inheritance [7]. Several genes have been identified in individuals affected by familial exudative vitreoretinopathy (FEVR). These genes encompass low-density lipoprotein receptor-related protein 5 (*LRP5*), frizzled-4 (*FZD4*), tetraspanin-12 (*TSPAN12*), zinc finger protein 408 (*ZNF408*), Norrie disease protein (*NDP*), catenin beta 1 (*CTNNB1*), exudative vitreoretinopathy 3 (*EVR3*), kinesin family member 11 (*KIF11*), atonal homolog 7 (*ATOH7*), RCC1 and BTB domain-containing protein 1 (*RCBTB1*), and jagged canonical Notch ligand 1 (*JAG1*). Significantly, the *FZD4*, *LRP5*, *TSPAN12*, and *NDP* genes play crucial roles in retinal vascular development through the Norrin-β-catenin (Wnt) signaling pathway, collectively accounting for approximately 90% of molecularly diagnosed FEVR cases. It's noteworthy that only 50% of FEVR cases feature identifiable variants, with the remaining 50% potentially linked to yet undiscovered loci or genes [8–12]. Therefore, the detection of these genetic variants is crucial for precise diagnosis and understanding of the underlying pathogenesis of the disease.

Although several studies have confirmed the pathogenicity of variants in FEVR patients using second-generation sequencing technologies and in silico analysis, few attempts have been made to analyze the conformational changes of protein structures at the atomic or molecular level. Molecular dynamics (MD) simulations have been utilized in other ophthalmologic studies to investigate the effects of mutant proteins on conformational dynamics [13–17], underscoring the significance of this approach in comprehending the functional mechanisms of proteins and the molecular underpinnings of diseases [18].

To explore the pathogenicity of FEVR variants at the dynamic molecular level, we combined targeted next-generation sequencing (TGS) panel with MD simulations and molecular docking to characterize novel pathogenic variants in nine Chinese families with clinically diagnosed FEVR. This approach aimed to provide additional evidence for understanding the molecular basis of FEVR.

## Methods

### Study subjects and clinical examinations

Clinical data were collected from patients with a confirmed diagnosis of FEVR who were treated at the Ningxia Eye Hospital and Ningxia Hui Autonomous Region People's Hospital. The study was conducted by the Declaration of Helsinki and approved by the Ethics Committee of Ningxia Hui Autonomous Region People's Hospital (No. 2020-KY-GZR019). Each participant or their legal guardians provided informed consent before participating in the study.

All probands and family members underwent comprehensive ophthalmic examinations, which included assessment of best corrected visual acuity (BCVA), slit-lamp biomicroscopy, funduscopy, ultra-widefield fundus photography (UWFFP, Daytona P200T, OPTOS, Fife, UK), and fundus fluorescein angiography (FFA; Spectralis HRA + OCT, Heidelberg Engineering GmbH, Heidelberg, DE). FEVR was diagnosed in patients presenting with avascular peripheral retina and neovascularization, brush-like vessels, RD, vitreous hemorrhage, severe subretinal exudates, and macular ectopia, as per the criteria outlined in previous studies [19]. The disease stage in patients with FEVR was determined for each eye using Trese's staging system [3]. Additionally, basic patient information, such as gender, age, birth history, and family history, was collected. Patients with a history of premature delivery or other retinal vascular diseases with similar clinical manifestations were excluded from the study.

### Pathogenic variant identification and in silico analysis

In this study, blood samples were collected from the proband and their family members to conduct TGS. Genomic DNA was extracted from peripheral blood leukocytes using the BaiMeng Magnetic Bead-based DNA Extraction Kit (BaiMeng, China). A whole-genome library was prepared using the NadPrep Whole-Genome Library Construction Kit (Swift Biosciences, USA) and selective enrichment of the exon regions of 511 target genes was performed using the SureSelect XT HS2 kit (Agilent Technologies, USA). Subsequently, paired-end 150-bp sequencing was performed on an Illumina HiSeq 2500 platform (Illumina, USA). The clean reads were aligned to the human reference genome hg19 (GRCh37) using the Burrows-Wheeler Aligner (BWA) software (Broad Institute of MIT and Harvard, USA). PCR duplicates were removed, and base quality score recalibration and variant calling were performed using the Genome Analysis Toolkit (GATK) software suite (Broad Institute, USA). The detected variants were annotated using the ANNOVAR software package (University of Michigan, USA) with data from various public databases, including dbSNP [20], ExAC [21], gnomAD [22], and 1000genomes [23]. Next, we excluded synonymous variants and non-coding region variants that had no impact on splicing signals. Additionally, variants with a high minor allele frequency of 0.01 or greater were also omitted from consideration. The pathogenicity and protein function of the variants were predicted using various tools such as SIFT [24], MutationTaster [25], PolyPhen-2 [26], REVEL [27], and GERP +  + [28, 29]. Each variant was further filtered in ClinVar [30] and HGMD [31] databases to confirm whether it was previously reported. Subsequently, mutations within *LRP5* (referenced by NM_002335.4 for mRNA) and *TSPAN12* (referenced by NM_012338 for mRNA) were validated and selected as candidate variants for further analysis.

Sanger sequencing was employed to validate the suspected variants identified within DNA segments of both probands and their family members, ensuring the verification of exon sequencing results. Specific primers tailored to encompass the potential variant sites within *LRP5* and *TSPAN12* were designed utilizing the Premier 5 software (PREMIER Biosoft, USA). These primers facilitated the amplification of DNA fragments harboring the pathogenic variant sites. Following amplification, these DNA fragments were incorporated into a PCR mixture comprising DNA primers, DNA polymerase, deoxyribonucleotide triphosphates (dNTPs, essential for DNA synthesis), and an appropriate buffer solution. The ensuing PCR process was cyclic, incorporating stages such as DNA denaturation, primer annealing, and DNA elongation. Post-PCR, the amplified products underwent purification via the QIAquick PCR purification kit (QIAGEN, Germany) and were then sequenced using the 3730xl Genetic Analyzer (Applied Biosystems, USA). Sequences derived from the family members were meticulously aligned against the target DNA fragment sequences from the probands. Using Chromas software (Technelysium Pty Ltd, Australia), these sequences, encompassing those of both the probands and their family members, were juxtaposed against a reference sequence, ensuring an accurate determination of the target region's DNA sequence.

Ultimately, co-segregation studies were conducted among the family members [32, 33], and the potential deleteriousness was assessed based on the American College of Medical Genetics and Genomics (ACMG) guidelines [34].

### Conservation analysis of LRP5 amino acids

The amino acid sequences of LRP5 proteins from various species were obtained from the universal protein resource (UniProt) [35] and analyzed for conservation among the species using ugene 48.1 software **(**Biomatters Ltd. New Zealand) for multiple sequence alignment. A phylogenetic tree was constructed to summarize the evolutionary and phylogenetic relationships among the species.

### The three-dimensional structure modeling

The three-dimensional (3D) structures of both the wildtype and mutations of LRP5 were generated using Alphafold2 [36] based on the amino acid sequence obtained from the UniProt website through multiple sequence alignment. The protein models were scored using the frame-aligned point error function (FAPE) [37], which is based on neural network training. The top-ranked result was selected to provide the structure for dynamic simulation, and the protein structure was visualized using PyMol 2.5.32 software [38]. The quality of the designed structure of the three proteins was evaluated using Ramachandran plots [39].

### Molecular dynamic simulation

To investigate the stability and conformational changes of the entire protein at the atomic level, we conducted a 200 ns MD simulation for both wildtype and mutant structures using the GROMACS 2019.6 software package (Department of Chemistry and Biotechnology, Sweden). The Amber14sb all-atom force field was chosen for MD simulations based on its extensive parameterization and its exceptional suitability for simulating complex macromolecular systems, including membrane proteins like LRP5 [40]. The wildtype and mutant protein structures underwent solvation in a transferable interatomic potential with three points (TIP3P) water model, in a cubic water box with a separation of 1 Å between the protein structure and the box edges, using the periodic boundary conditions to avoid edge effects [41, 42]. The wildtype system required 98 Na + ions for neutralization, compared to 58 for p.Trp691Cys and 55 for p.Pro1431Argfs*8 mutants. Then relaxed by the steepest descent algorithm and the conjugate gradient algorithm within 50,000 steps. The coulomb force and van der Waals interactions were calculated based on a cut-off distance of 1.4 nm. The protein topology was established following the aforementioned steps and then equilibrated using the NVT (isochoric-isothermal) and NPT (isothermal-isobaric) ensembles [43]. Subsequently, a 200 ns MD simulation was conducted, during which data on root mean square deviation (RMSD), root mean square fluctuation (RMSF), and radius of gyration (RG) were collected for subsequent analysis. Hydrogen bond number (HBNUM) and secondary structure calculations were carried out by the dictionary secondary structure pattern (DSSP) algorithm [44]. Snapshots of the system were captured at regular intervals of 50 picoseconds for subsequent analyses. Trajectories of the C-α atoms were plotted employing Origin 85 software (OriginLab Corporation, USA). Furthermore, to elucidate the dynamic conformational changes in the three proteins, we generated graphical representations as well as video clips to visualize the structural variations throughout the entirety of the MD simulation. These visualizations were performed using PyMOL 2.5.32 software platforms. Accompanying video materials are provided in Additional File 1.

### Molecular docking

DKK1 is a pivotal protein involved in the regulation of the Wnt signaling pathway. As a member of the Dickkopf protein family, it acts as a Wnt antagonist. DKK1 exerts its function by binding to the LRP5 co-receptors, thereby impeding Wnt signaling activation and suppressing the canonical Wnt pathway [45]. The AlphaFold-predicted 3D model of DKK1 was acquired from the UniProt website and subsequently processed using PyMOL version 2.5.32. This processing involved the removal of water molecules, hydrogen atoms, and extraneous protein regions.

In the docking experiments, ZDOCK version 3.0.2 [46] served as the computational tool for executing global rigid docking. The crystal structure, identified by PDB ID 5GJE, provided a reference framework for establishing the interaction interface between DKK1 and LRP5. Specifically, the C-terminal region of DKK1 (residues 178–266) was anchored to the highly conserved third and fourth YWTD-EGF-β-propeller domains (residues 631–1246) of LRP5. Subsequent docking involved aligning the N-terminal region of DKK1 with the first and second YWTD-EGF-β-propeller domains of LRP5.

Upon completing the docking, a 30 ns MD simulation was conducted using GROMACS 2019.6 and the Amber14sb all-atom force field, by the procedures detailed in the "Molecular Dynamics Simulation" section. Subsequently, binding free energy was calculated employing the MM/GBSA method [47, 48]. Trajectory snapshots were saved at 10 ps intervals for clustering analysis. The structure with the highest clustering frequency was then scrutinized for binding modes via PyMOL version 2.5.3.

### Alanine scanning mutagenesis

Alanine scanning is a widely used technique for investigating the role of specific amino acid residues in a protein's function. By replacing these residues with alanine, which has minimal structural impact due to its small, nonpolar nature, the technique allows for an assessment of the consequences of missense variants. It also contributes to our understanding of the functional significance of each residue [49]. To analyze the local residue changes and binding free energies resulting from alanine mutations, we employed alanine scanning by manually introducing alanine mutations at positions 691 and 1431 of the wildtype LRP5 protein. Subsequently, we conducted 30 ns MD simulations and calculated binding free energies for the complex between the alanine mutant protein and DKK1, using the methodology described in the docking section.

### Statistical analysis

The comparison between the wildtype and two mutant proteins (p.Trp691Cys and p.Pro1431Argfs*8) was performed using Tukey's method with SPSS 26.0 software (IBM Corporation, USA). Statistical significance was determined based on a threshold *P*-value of less than 0.05.]

**Fig. 1:**
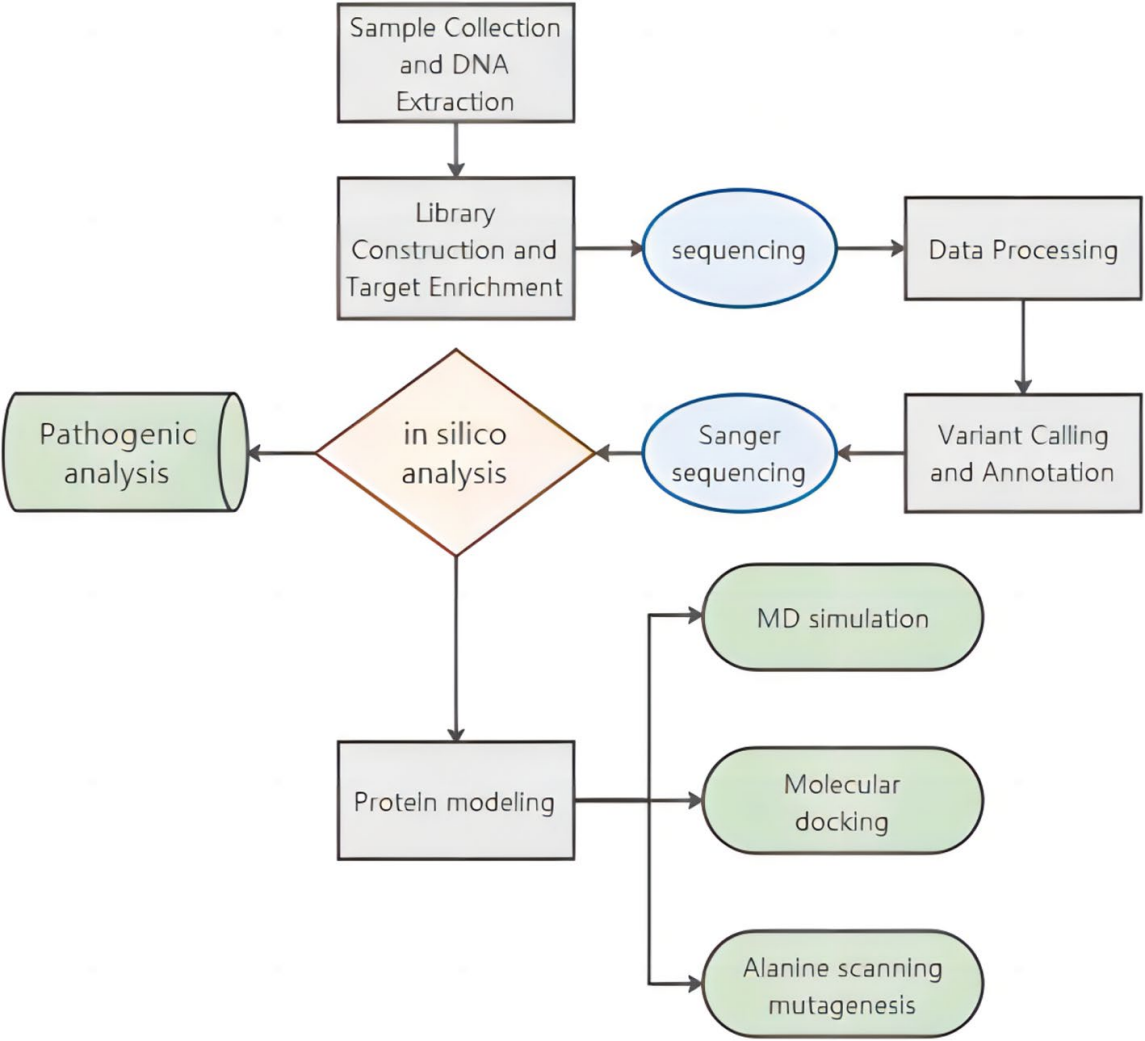
The flowchart illustrates the sequencing and computer simulation processes

To elaborate on the methodology, we created a flowchart to illustrate the experimental process, see Fig. 1.

## Results

### Clinical manifestations

**Fig. 2:**
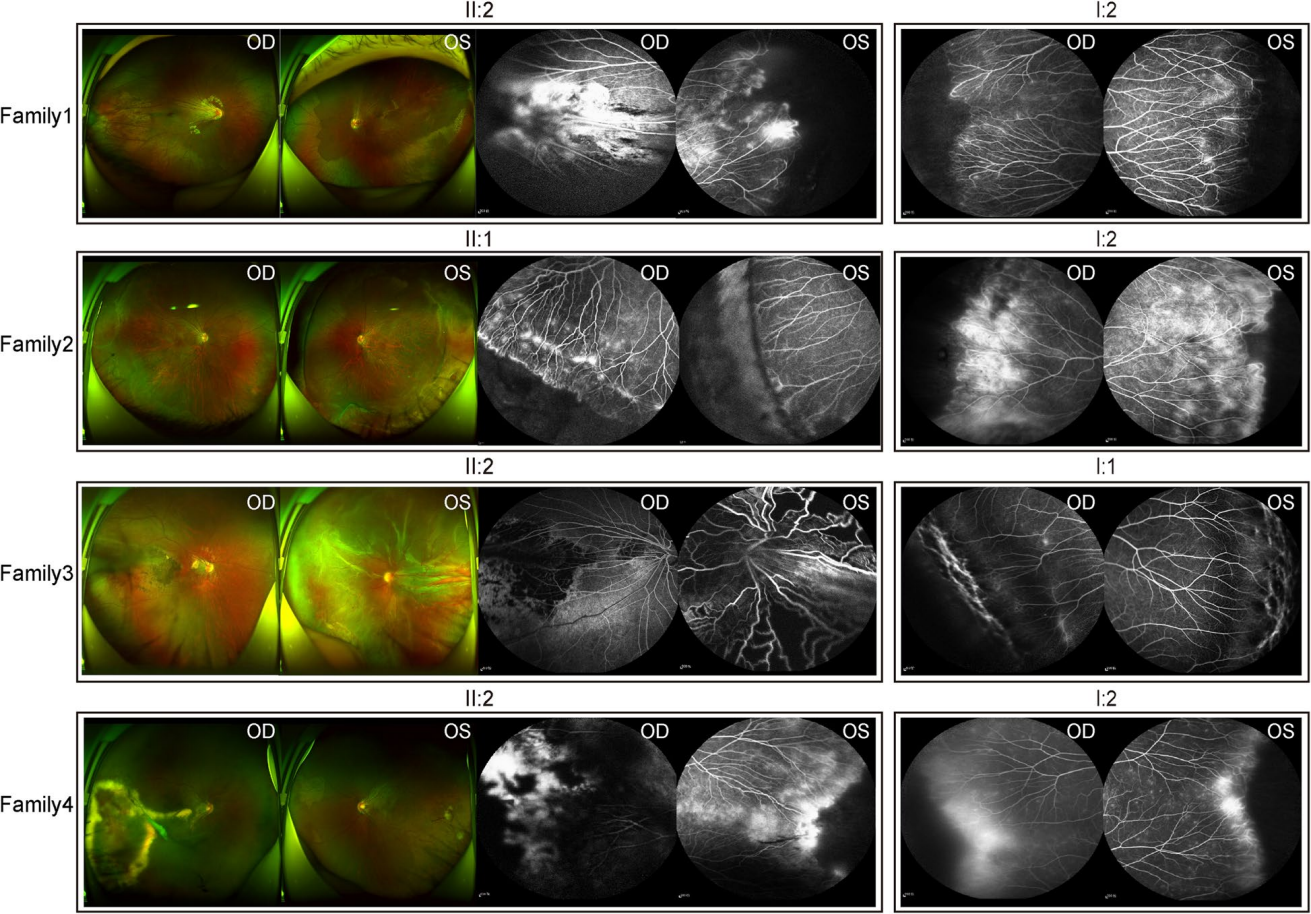
Fundus Photography and Fluorescein Angiography of Individuals Affected by FVER. Fundus photographs and fluorescein angiography images of probands in four different families are presented as increased vascular branches, fluorescein leakage, no perfusion area scleral buckling or paraoptic disc retinal atrophy, retinal detachment, while their parents only showed no perfusion area and increased vascular branches or fluorescein leakage. Abbreviations: FVER stands for familial exudative vitreoretinopathy, OD for the right eye, and OS for the left eye

The general data, visual acuity results, fundus photographs, and FFA of eight affected individuals, including the proband and first-degree relatives from four families were shown in Table 1. The probands consisted of three males and one female, with a mean age of 12.25 years (range, 7 to 17 years), while the affected family members included one male and three females, with a mean age of 43.25 years (range, 42 to 45 years). BCVA ranged from finger count before the eye to 1.0. Notably, fundus manifestations varied significantly among individuals, even within the same family. 3 out of 4 probands were categorized as stage 3, while their parents were classified as stage 2. Specifically, the FFA of the probands from three families exhibited RD in either of the eyes, except for the proband from family 1, whose fundus only showed dilated blood vessels and fluorescein leakage. In contrast, their parents consistently showed no perfusion area, increased vascular branching, and stained vascular walls (Fig. 2).

**Table 1:** Summary of general information and phenotype characteristics of affected individuals from 4 FEVR families

Family	Patients	Gender	Age at Initial visit	BCVA		FEVR Stage		FFA
				**OD**	**OS**	**OD**	**OS**	
Family 1	II2:	F	9y	0. 1	0. 4	2	2	OU: Dilated blood vessels; fluorescein leakage
	I2:	F	42y	1. 0	1. 0	2	2	OU: no perfusion area; increased vascular branches; stained vascular wall
Family 2	II1:	M	17y	1. 0	0. 6	2	3	OD: fluorescein leakage; no perfusion area OS: postoperative of RD
	I2:	F	45y	0. 8	0. 8	2	2	OU: no perfusion area; increased vascular branches
Family 3	II2:	M	7y	0. 2	FC/BE	2	3	OD:paraoptic disc and temporal retinal atrophy, fluorescein leakage OS: RD and retinal fold
	I1:	M	43y	1. 0	1. 0	2	2	OU: no perfusion area; increased vascular branches; stained vascular wall
Family 4	II2:	M	16y	0. 5	0. 7	3	2	OD: RD and fluorescein leakage OS: no perfusion area; increased vascular branches; fluorescein leakage
	I2:	F	43y	1. 0	1. 0	2	2	OU: no perfusion area; increased vascular branches; fluorescein leakage

*Abbreviations*: *FEVR* familial exudative vitreoretinopathy, *RD* retinal detachment, *F* female, *M* male, *OD* right eye, *OS* left eye, *OU* both eyes, *BCVA* best corrected visual acuity, *FC/BE* finger count before the eye, *FFA* fundus fluorescein angiography

### Variants detected by targeted genetic sequencing

**Fig. 3:**
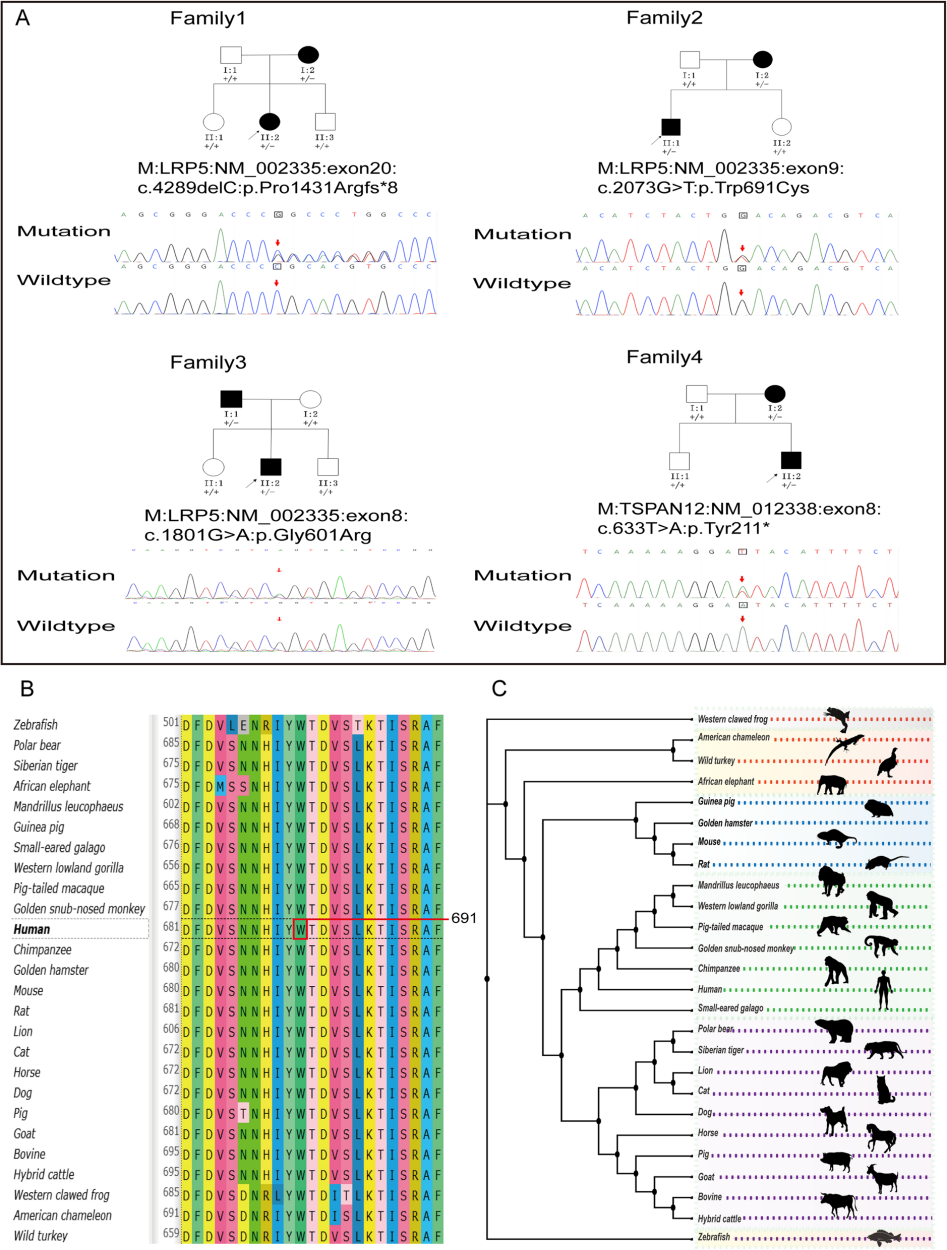
Genetic findings in four families and conservative analysis of a novel missense variant. **A** Pedigrees of the families with causative variants and the corresponding Sanger sequencing results of a heterozygous variant in the proband and his/her first-degree non-affected relative. Squares represent males, circles represent females, arrows indicate the proband, open symbols represent unaffected individuals, solid symbols represent affected individuals, ± represents heterozygous variant, and + / + represents wildtype. **B** Alignment of LRP5 homologous protein sequences from 26 species, using human LRP5 as a reference, reveals a remarkable conservation of the tryptophan residue at position 691 across these species. **C** The phylogenetic trees represent the evolutionary relationships of various species

Four variants were detected in 4 out of 9 (44%) families (Table 2, Fig. 3A). Among these, two novel heterozygous variants (c.4289delC: p.Pro1431Argfs*8 and c.2073G > T: p.Trp691Cys) were identified in *LRP5*, while the other two variants (c.1801G > A: p.Gly601Arg in *LRP5* and c.633 T > A: p.Tyr211* in *TSPAN12*) had been previously reported [50, 51].

**Table 2:** Computational analysis of variants in four probands with FEVR

Family	proband	Gene	ORF and Amino acid changes	Allele Status	MAF	SIFT	Polyphen-2	REVEL	Mutation Taster	GERP + +	ACMG	Genetic Model	Ref
Family 1	II2	*LRP5* NM_002335.4: exon20	c.4289delC: p.Pro1431Argfs*8	Het	NA	NA	NA	NA	DC	4. 53	Pathogenic	AD	Novel
Family 2	II1	*LRP5* NM_002335.4: exon9	c.2073G > T: p.Trp691Cys	Het	NA	D	PrD	0. 994	DC	4. 11	Pathogenic	AD	Novel
Family 3	II2	*LRP5* NM_002335.4: exon8	c.1801G > A: p.Gly601Arg	Het	0. 0000083	D	PrD	0. 965	DC	4. 13	Pathogenic	AD	Reported
Family 4	II2	*TSPAN12* NM_012338: exon8	c.633 T > A: p.Tyr211*	Het	NA	NA	NA	NA	DC	5. 68	Pathogenic	AD	Reported

In silico analyses: SIFT, Polyphen-2, REVEL, Mutation Taster, and GERP +  +

*Abbreviations*: *FEVR* familial exudative vitreoretinopathy, *ORF* open reading frame, *MAF* minimum allele frequency, *ACMG* American College of Medical Genetics and Genomics, *Ref* reference, *Het* heterozygous, *D* damaging/ deleterious, *DC* disease-causing, *PrD* probably damaging, *NA* not available, *AD* autosomal dominant inheritance

The newly identified variant c.4289delC: p.Pro1431Argfs*8 in *LRP5* caused a Pro to Arg substitution at position 1431, followed by premature termination at position 1437 due to eight frameshift amino acids. This resulted in a truncated protein product. The second novel variant, c.2073G > T: p.Trp691Cys, was found in family 2 and led to a Trp to Cys substitution. These two variants were absent in other healthy family members but were detected in the probands and affected relatives.

Two variants, c.1801G > A: p.Gly601Arg in *LRP5* and c.633 T > A: p.Tyr211* in *TSPAN12*, were identified in families 3 and 4, respectively, and have been previously reported. The c.1801G > A missense variant in *LRP5* resulted in the substitution of Gly with Arg at position 601, while the c.633 T > A missense variant in *TSPAN12* led to a nonsense mutation at Tyr position 211, resulting in protein truncation.

### Pathogenicity analysis

While sequencing validation was not conducted in the control population, the two variants in the *LRP5* gene (c.4289delC: p.Pro1431Argfs*8 and c.2073G > T: p.Trp691Cys), as well as one variant in the *TSPAN12* gene (c.633 T > A: p.Tyr211), were not identified in public databases such as dbSNP, ExAC, or 1000 Genomes. In contrast, the minimum allele frequency (MAF) of the c.1801G > A: p.Gly601Arg variant was 0.0000083.

In silico analysis methods SIFT, Polyphen-2, REVEL (scored 0.994, and 0.965 respectively), MutationTaster, and GERP +  + (scored 4.11, and 4.13 respectively) predicted two of the variants (c.2073G > T: p.Trp691Cys and c.1801G > A: p.Gly601Arg) to be disease-causing or damaging. The remaining two variants (c.4289delC: p.Pro1431Argfs*8 and c.633 T > A: p.Tyr211*) were predicted to be disease-causing or damaging by MutationTaster and GERP +  + (scored 4.53 and 5.68 respectively) (Table 2). All four variants exhibited co-segregation among affected family members. Notably, the newly identified missense variant c.2073G > T: p.Trp691Cys is evolutionarily conserved across a broad spectrum of species, extending from humans to zebrafish (Fig. 3B and C). Therefore, all these variations were considered pathogenic according to the ACMG standard.

### Protein structure evaluation

**Fig. 4:**
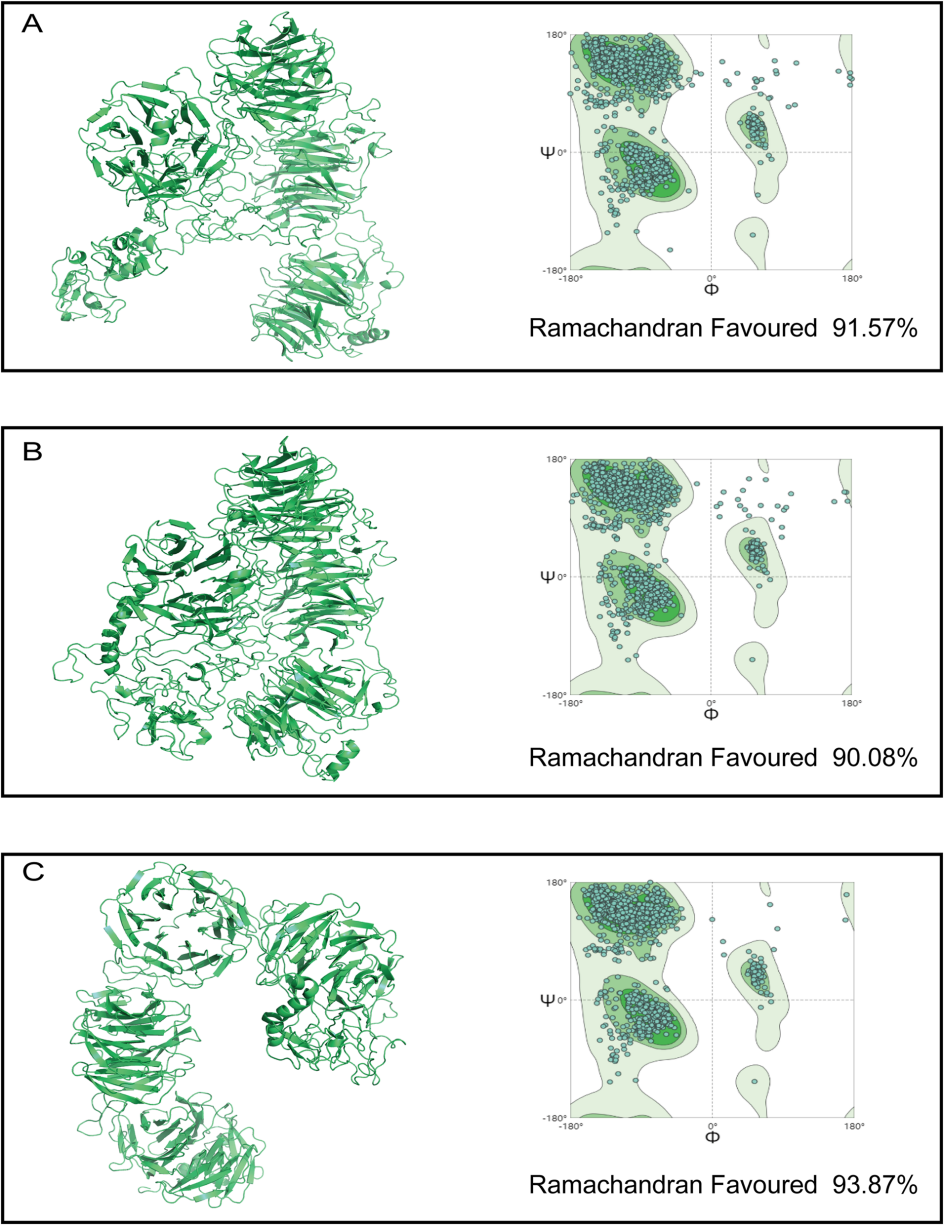
The protein structures were modeled using AlphaFold2 and their conformations were evaluated via Ramachandran plots. **A** For the wildtype protein, 91.57% of amino acid residues were found in the favored regions. **B** In the p.Trp691Cys mutant, 90.08% of residues were in the favored regions. **C** The p.Pro1431Argfs*8 mutant showed 93.87% of its residues in the favored areas

The Ramachandran plot analysis of the three structures in Fig. 4 reveals that amino acid residues falling within the favored regions make up more than 90% of the entire protein. This suggests that the models' conformations conform to the fundamental principles of stereochemistry [52].

### Molecular dynamic simulation and data analysis

**Fig. 5:**
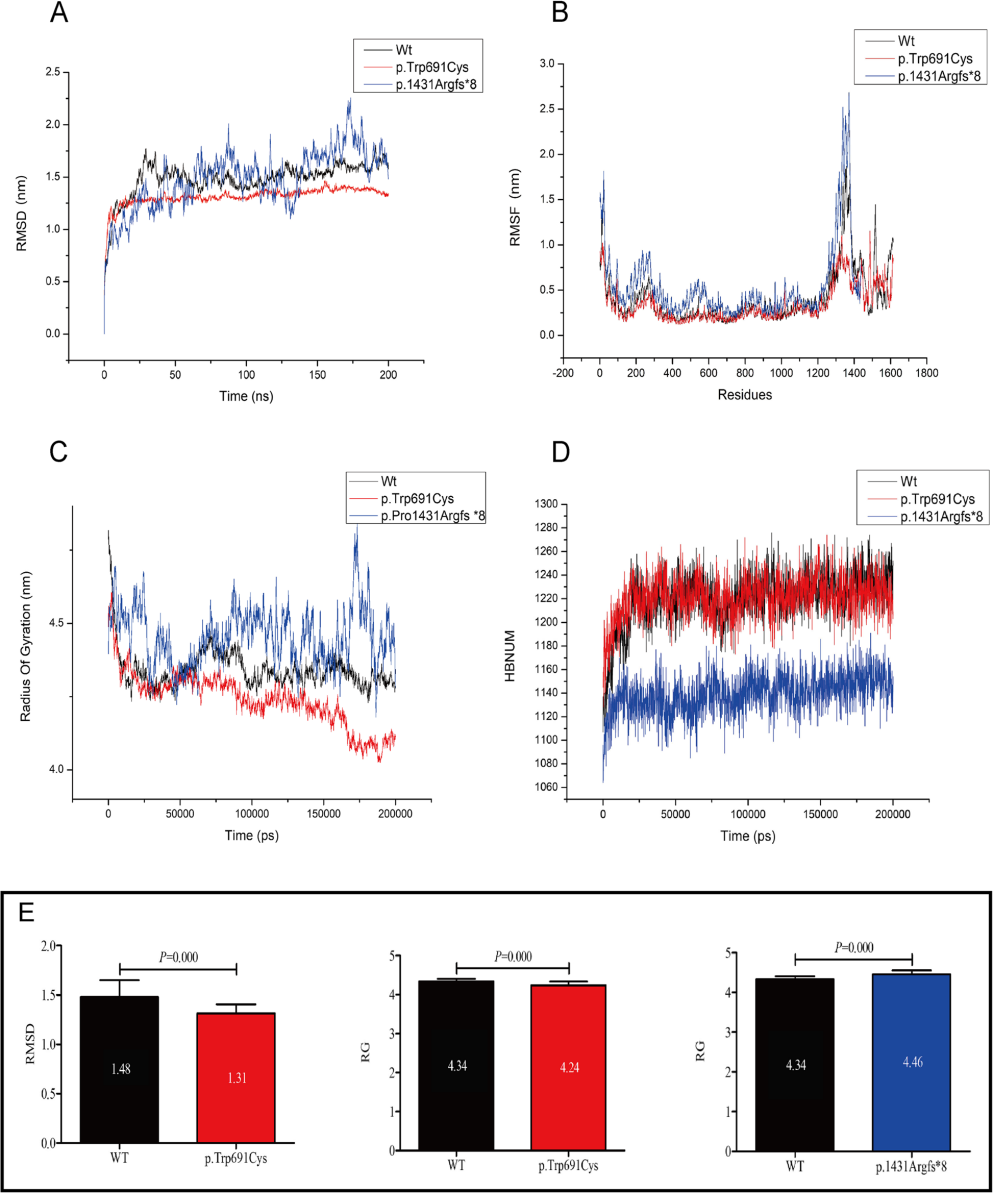
RMSD, RMSF, Rg, HBNUM values comparisons between wildtype and two mutant proteins in MD simulation. **A**-**D** Evolution over time to show RMSD, RMSF, Rg, and HBNUM values of the wildtype (black) and the two mutant proteins (p.Trp691Cys in red, p.Pro1431Argfs *8 in blue). **E** Statistical analysis comparison of RMSD and RG values as bar graphs, depicting values with *p* < 0.05 (95% CI)

RMSD and RMSF have commonly used measures to assess the spatial variations of proteins in MD simulations. RMSD quantifies the overall difference of a molecule compared to a reference conformation. The RMSD values of the wildtype and two LRP5 mutations were illustrated in Fig. 5A. During the simulation, the p.Trp691Cys variant showed smaller conformational changes than the wildtype (*P* = 0.000, < 0.05, 95%CI) (Fig. 5E), while the p.Pro1431Argfs*8 variant did not show statistically significant differences in conformational changes compared to the wildtype (*P* > 0.05, 95% CI). In the end, the backbone atoms of all three proteins remained around 1.5 nm.

**Fig. 6:**
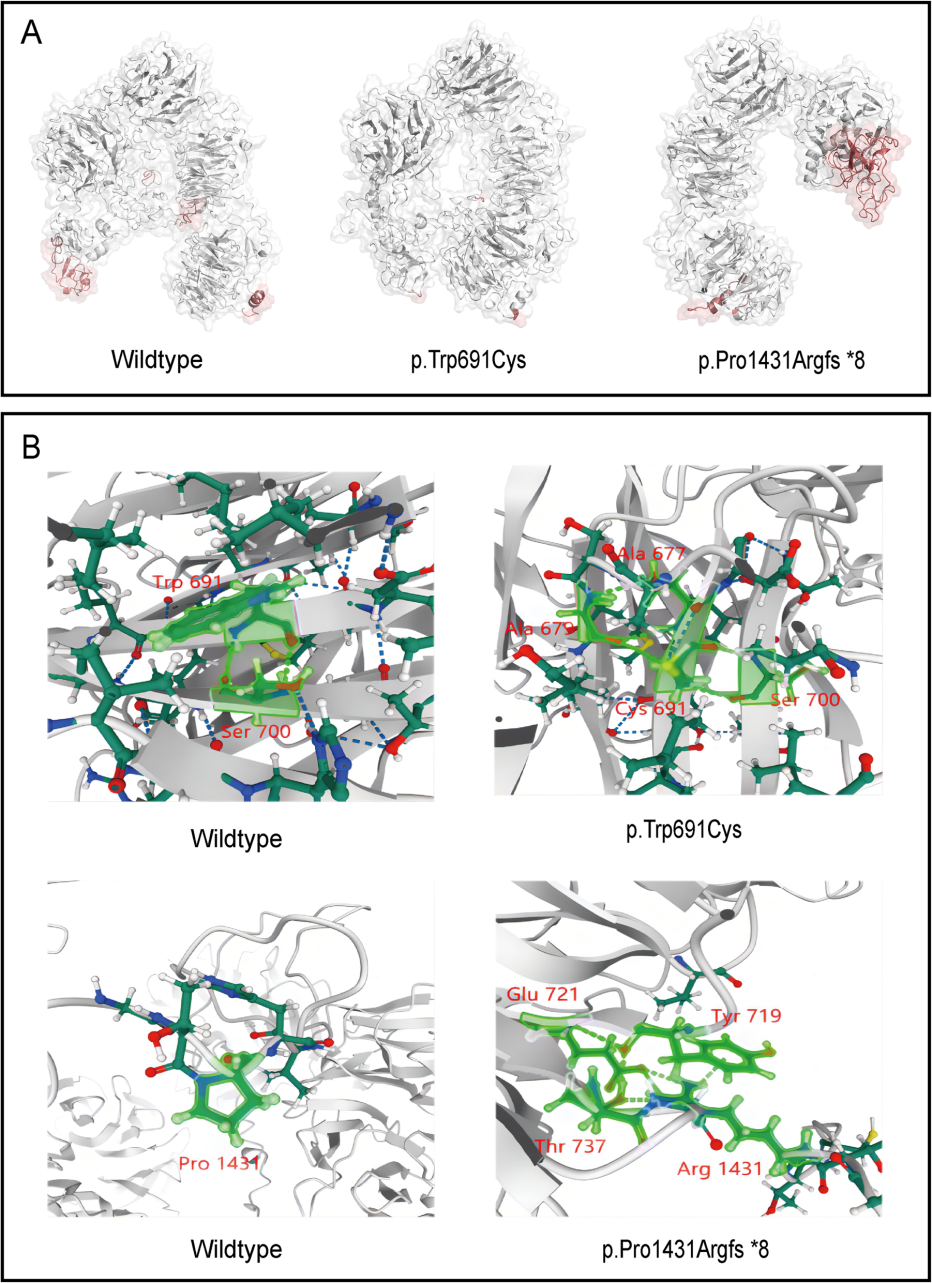
Comparison of active sites and H-bonds between the wildtype and mutant proteins. A. Changes in active sites in wildtype and two mutant proteins during simulation were marked in red. B. The snapshots extracted from the last frame of the MD simulation showed the intramolecular H-bonds changes in wildtype and mutants. The target residues along with H-bonds were marked in bright green

The RMSF analysis revealed distinct flexibility profiles among the wildtype and mutant proteins at specific residues (Fig. 5B) The wildtype's most flexible regions were Trp10-Ala27, Asp1318-Ile1387, Met1513-Asn1520, and Pro1606-Ser1615. In contrast, p.Trp691Cys displayed limited flexibility at Leu14-15, Asp1331-1333, and Ser1486-Thr1489; p.Pro1431Argfs8 had flexibility at Met1-Pro28 and Pro1261-Ser1385. Average RMSF values for the active sites fluctuated between 1.03–1.38 nm, 1.02–1.08 nm, and 1.42–1.53 nm for the wildtype, p.Trp691Cys, and p.Pro1431Argfs8, respectively. Maximum RMSF values were 1.87 nm, 1.16 nm, and 2.68 nm, respectively, implying reduced conformational changes in p.Trp691Cys and heightened local activity in p.Pro1431Argfs8, especially at Met1-Pro28 and Pro1261-Ser1385 (Fig. 6A). These findings suggest that the pathogenic variants may influence the flexibility of the protein backbone, which could affect the protein's structure and function. Especially evident in the videos of Additional file 1, the conformational changes of the three proteins over time were well-illustrated. Although the C-terminal region of the p.Pro1431Argfs*8 mutant exhibited heightened activity, it too reached a state of convergence by the end of the simulation.

Rg values, which measure the distance or fluctuation from C-α atoms to the center of mass, were 4.34 nm, 4.25 nm, and 4.45 nm for the wildtype and two mutants, respectively (Fig. 5C). The C-α atoms in the p.Pro1431Argfs*8 mutant showed greater fluctuation (*P* = 0.000, < 0.05, 95%CI)) compared to the wildtype, while the p.Trp691Cys mutant exhibited less fluctuation (*P* = 0.000, < 0.05, 95% CI) (Fig. 5E). These results suggest that the p.Trp691Cys variant may increase the rigidity of the protein, whereas the p.Pro1431Argfs*8 variant may enhance its flexibility at the local conformation, especially in the regions of Met1-Pro28 and Pro1261-Ser1385.

HBNUM stands for the number of hydrogen bonds (H-bond), which are crucial interactions for stabilizing the spatial arrangement of proteins. It is evident from the results that the number of H-bonds in p.Trp691Cys is similar to that of the wildtype, while in p.Pro1431Argfs*8, which is a truncated protein due to the absence of C-terminus structures, the overall number of H-bonds is reduced compared to the wildtype (Fig. 5D). In terms of intramolecular interactions through H-bonds, as shown in Fig. 6B, Trp691 formed H-bonds with Ser700 in the wildtype, while the Cys691 of p.Trp691Cys formed H-bonds with Ala677, Ala679, and Ser700. In the p.Pro1431Argfs*8 mutant, the Pro1431 was transformed into Arg, which formed H-bonds with Tyr719, Glu721, and Thr737. The changes in H-bond distribution certainly contributed to the structural and conformational alterations.

**Fig. 7:**
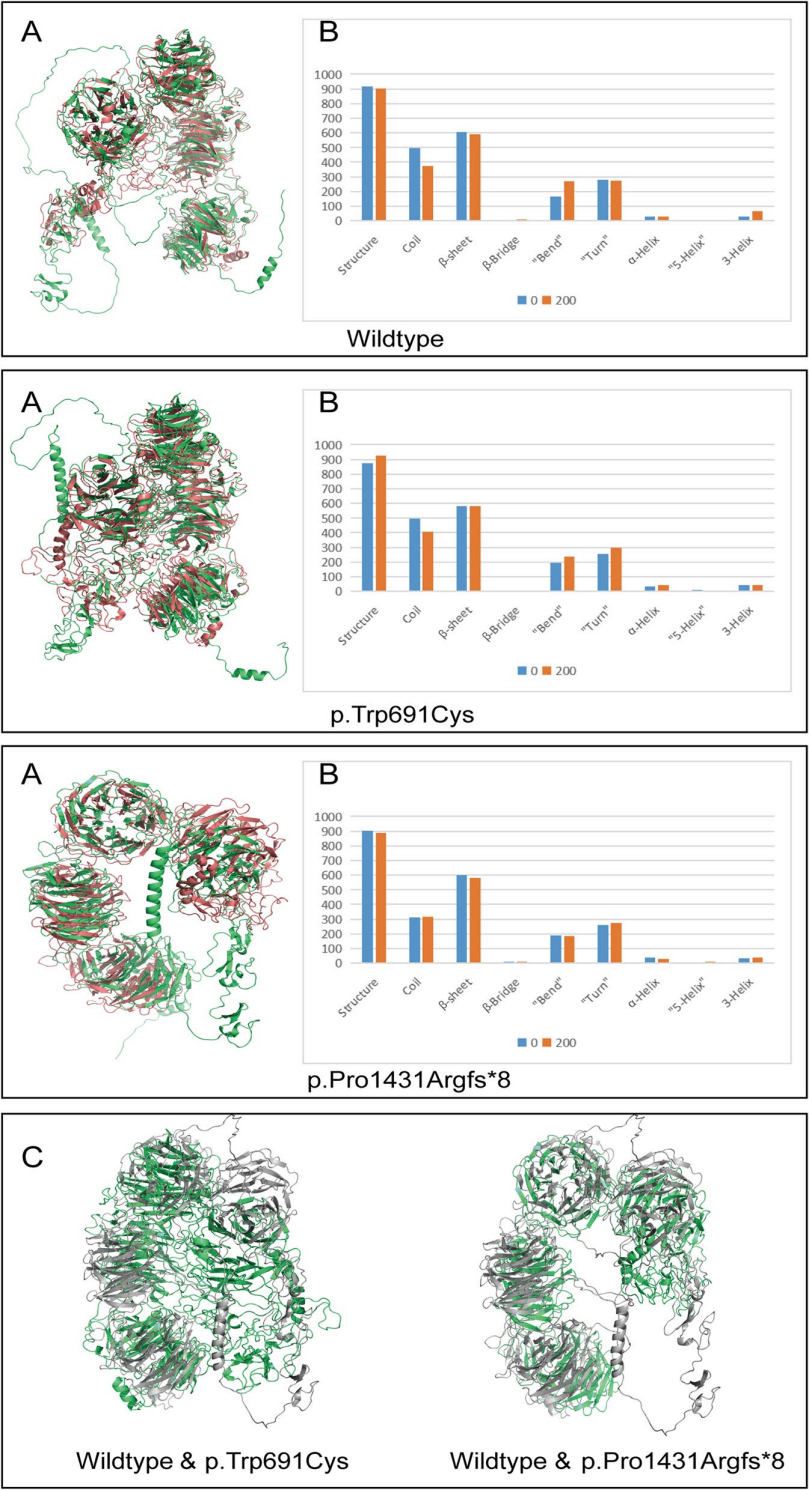
Molecular Trajectories and Structural Comparisons of Wildtype and Mutant Proteins in MD Simulation. **A** Snapshots of molecular trajectories for the three proteins at both the initial and final frames of the simulation are superimposed and color-coded, with the initial frame marked in green and the final frame in red. **B** Quantitative analysis of eight principal secondary structures (coil, β-sheet, β-bridge, bend, turn, α-helix, 5-helix, and 3-helix) in the wildtype and the two mutants, p.Trp691Cys and p.Pro1431Argfs*8. **C** The figure displays snapshots of molecular trajectories at the final frame of the simulation, comparing the wildtype and two mutant proteins. The wildtype protein is marked in grey, while the two mutant proteins are denoted in green

To perform DSSP analysis, the last 50 ps of the simulation were selected, and the final frames of the MD simulations for the three proteins were obtained and compared with their respective initial structures. Additionally, a comparison was made between the 3D structures of the wildtype and the two mutant proteins in the final frame. All three ensembles were observed to converge to more stable conformations relative to their initial states, as depicted in Fig. 7A. In the final frame, both mutant proteins exhibited greater structural convergence and rigidity compared to the wildtype protein (Fig. 7C).

Figure 7B presents the DSSP analysis results. Post-simulation, the wildtype protein showed a decrease in coil, β-sheet, and turn structures, while witnessing an increase in bend and 3-helix formations. The α-helix configuration remained constant, and a β-bridge structure emerged. Importantly, the 5-helix structure was absent throughout the entire simulation period.

Before the simulation, p.Trp691Cys had fewer β-sheet and turn structures than the wildtype but more bend, 3-helix, and α-helix structures. Post-simulation, turn and α-helix structures increased, surpassing the wildtype, while β-sheet remained stable. The β-bridge disappeared, and no 5-helix was observed at any stage.

For p.Pro1431Argfs*8, pre-simulation levels of β-sheet were similar to the wildtype, but turn structures were less abundant. Bend, 3-helix, and α-helix were more prevalent. Post-simulation, turn structures increased to wildtype levels; bend and α-helix decreased while 3-helix rose, still lagging behind the wildtype. A 5-helix structure appeared post-simulation. Throughout, the coil structure was consistently less abundant than the wildtype. The DSSP analysis is visually represented in Additional File 2.

### Molecular interactions

In the docking simulation, the binding energies for wildtype-DKK1, p.Trp691Cys-DKK1, and p.Pro1431Argfs*8-DKK1 were -90.37 ± 2.35 kcal/mol, -135.78 ± 2.75 kcal/mol, and -118.69 ± 5.38 kcal/mol, respectively. Residue decomposition showed that hydrophobic and electrostatic interactions mainly drove binding, followed by nonpolar solvation-free energy. Both mutant proteins displayed larger negative values for van der Waals and electrostatic energies compared to the wildtype, indicating a stronger affinity for DKK1. Solvation factors for the mutants also suggested a more favorable binding environment with DKK1 (Table 3). These results indicate that both p.Trp691Cys-DKK1 and p.Pro1431Argfs*8-DKK1 have higher binding affinity compared to wildtype-DKK1 protein. Therefore, the pathogenic variants enhance the binding propensity of the protein with DKK1.

**Table 3:** Binding free energies and energy components predicted by MM/GBSA (kcal/mol)

System name	wildtype-DKK1	p.Trp691Cys-DKK1	p.Pro1431Argfs*8-DKK1
Δ*E*_vdw_	-161.88 ± 4.45	-256.31 ± 5.50	-261.36 ± 5.55
Δ*E*_elec_	-3151.37 ± 21.50	-4545.09 ± 40.48	-4528.62 ± 36.38
ΔG_GB_	3245.26 ± 21.90	4700.90 ± 44.12	4708.74 ± 36.94
ΔG_SA_	-22.37 ± 0.69	-35.27 ± 0.64	-37.44 ± 0.88
ΔG_bind_	-90.37 ± 2.35	-135.78 ± 2.75	-118.69 ± 5.38

*ΔEvdW* van der Waals energy

*ΔEelec* electrostatic energy

*ΔGGB* electrostatic contribution to solvation

*ΔGSA* non-polar contribution to solvation

*ΔGbind* binding free energy

**Fig. 8:**
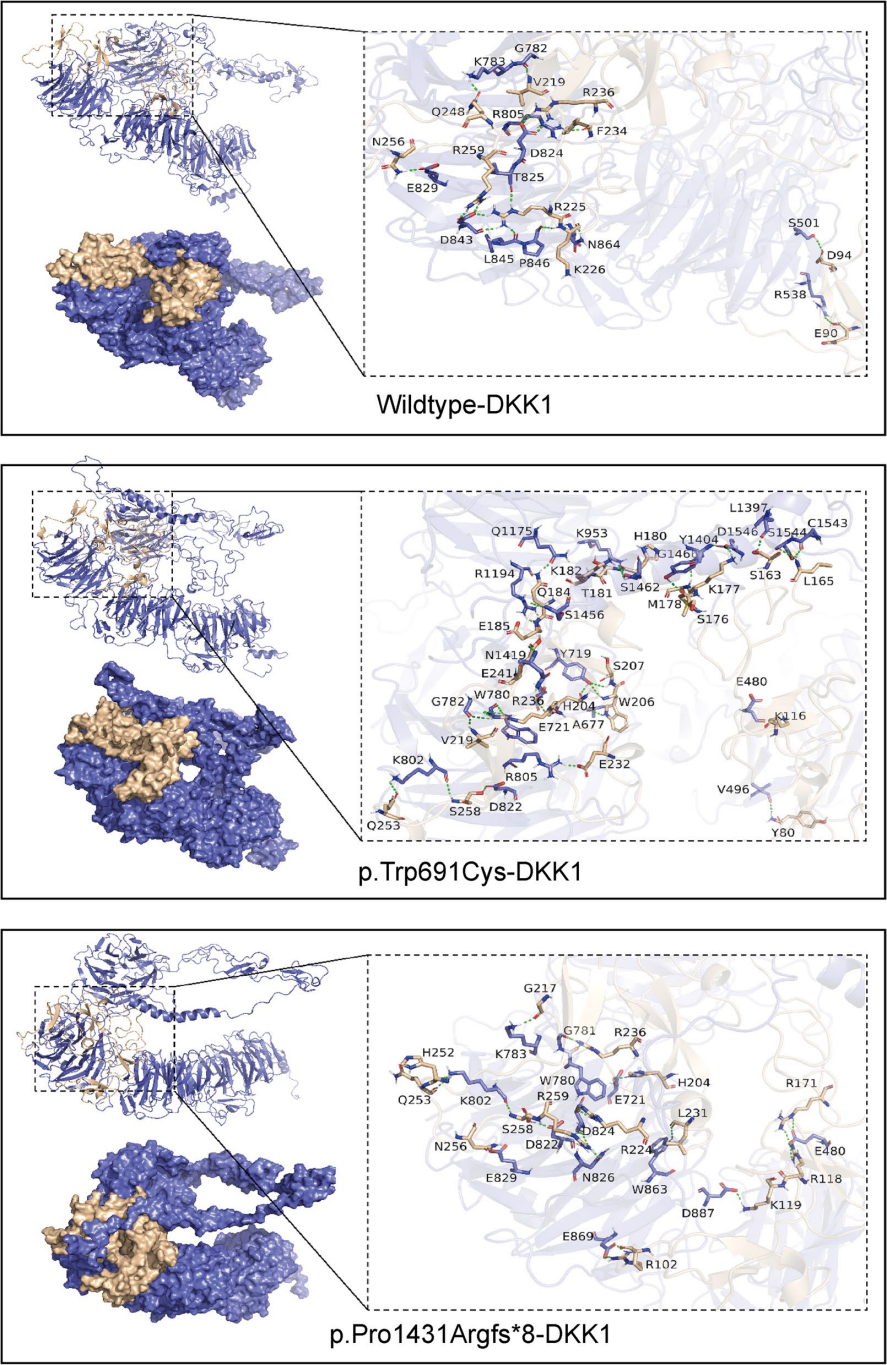
The 3D binding modes of the wildtype, p.Trp691Cys, and p.Pro1431Argfs*8 systems with DKK1 protein are shown, with a focus on the docking site. The LRP5 protein is represented in purple, while the DKK1 protein is depicted in a yellow–brown color

Figure 8 illustrates the H-bond interactions between DKK1-C (residues 178–266) and the conserved third and fourth YWTD-EGF-β-propeller domains (residues 631–1246) of LRP5. Specifically, nine residues on DKK1 form H-bonds with nine residues on the wildtype protein.

In the p.Trp691Cys-DKK1 complex, 11 residues from each protein are involved in H-bond formation. Similarly, in the p.Pro1431Argfs*8-DKK1 complex, 10 residues from each protein contribute to hydrogen bonding. These interactions provide insight into the differential affinities and stabilizing factors in the binding of wildtype and mutant LRP5 to DKK1.

Compared to the wildtype, both the p.Trp691Cys and p.Pro1431Argfs*8 mutants exhibit an increased number of H-bonds in their protein binding complexes, along with a change in the residue types that interact with DKK1 protein. Details on the specific H-bond interactions and bond lengths can be found in Additional File 3.

### Alanine mutagenesis analysis

By comparing the mutated residues with alanine residues, the impact of point mutations on protein structure is elucidated. As shown in Additional File 4, the alanine variants at positions 691 and 1431 lead to a decrease in binding free energies and an increase in binding affinity compared to the wildtype. The binding free energy for the p.Trp691Ala is -134.75 ± 3.96 kcal/mol, while the binding free energy for the p.Pro1431Ala is -95.54 ± 3.18 kcal/mol. In contrast, the wildtype binding free energy is -90.37 ± 2.35 kcal/mol.

## Discussion

In this study, 3 variants of *LRP5* and 1 of *TSPAN12* in four unrelated FEVR families were found to be pathogenic according to the ACMG guidelines. Two of the *LRP5* variants, c.4289delC (p.Pro1431Argfs*8) and c.2073G > T (p.Trp691Cys), were novel variants with extensive changes in protein structure according to MD simulation. While the other two variants, c.1801G > A (p.Gly601Arg) in *LRP5* and c.633 T > A (p.Tyr211*) in *TSPAN12*, have been previously reported [50, 51]. It is reported that approximately 50% of FEVR patients harbored deleterious variants [3], among which *LRP5* accounts for about 12%—25% and *TSPAN12* for about 3%—10% [8, 53–56], and that the mutation frequency of the same gene varies in different ethnicities. In this study, the total detection rate of all variants was 44% (4/9), which was consistent with previous reports [3]. Of all the FEVR patients, 75% (3/4) were caused by the *LRP5* variant, whereas 25% (1/4) were by the *TSPAN12* variant. However, Zou. et, al. [51] observed that in the same region of China (Ningxia), four out of eight probands (50%, 4/8) harbored pathogenic variants in *TSPAN12* (75%, 3/4) and *FZD4* (25%, 1/4) genes, but none of them carried *LRP5* variants. Combining the data from the two research revealed that in Ningxia, *LRP5* accounted for 37.5% (3/8) and *TSPAN12* for 50% (4/8) of FEVR patients. As a result, the *LRP5* and *TSPAN12* genes were therefore hypothesized to be the more prevalent causative genes in the Ningxia population. Certainly, it is still necessary to verify these findings with a larger sample size to rule out the possibility of selection bias in subsequent investigations. Due to the absence of any literature indicating gender differences in this disease, we did not investigate gender disparities in our study.

As previously documented, variants in *LRP5*, exhibiting autosomal dominant, recessive, and X-linked inheritance patterns, lead to disturbances in the Wnt signaling pathway. These disruptions, in turn, result in aberrant retinal vascular development, giving rise to the onset of lesions associated with familial exudative vitreoretinopathy (FEVR) [57, 58]. Additionally, it has been reported that the recessive inheritance pattern of *LRP5* not only causes abnormalities in retinal vascular development but also gives rise to severe skeletal anomalies [58]. In this study, we identified three pathogenic variants of *LRP5* with an autosomal dominant inheritance pattern, among which the novel missense variant c.2073G > T (p.Trp691Cys) was found. This variant is located in the highly conserved third YWTD-EGF-β-propeller domains. Studies of *LRP6* suggest that the third YWTD-EGF repeats are the region to bind with ligands of the Wnt signal pathway [45]. Considering the high similarity between LRP5 and LRP6 proteins [45], It is plausible to hypothesize that the c.2073G > T (p.Trp691Cys) variant in *LRP5* could potentially impact the Wnt signaling pathway by influencing the binding of extracellular ligands to the YWTD-EGF-β-propeller domains. Another frameshift variant of *LRP5*, c.4289delC (p.Pro1431Argfs*8), was first identified in this study, which resulted in a premature termination of the protein at position 1437 and generated a truncated protein that may be pathogenic due to the loss of function.

To elaborate the pathogenic mechanism at the dynamic molecular level, we employed alphafold2 to model the 3D structure of LRP5 for the first time and implemented 200 ns MD simulation to analyze the changes in RMSD, RMSF, RG, HBNUM, and DSSP values in wildtype and mutant proteins. These indicators suggest that the p.Trp691Cys and p.Pro1431Argfs*8 variants may have triggered FEVR manifestation by modifying the overall conformation of the LRP5 protein, potentially affecting its ligand binding. Numerous studies and experiments have established that the flexibility of specific regions in proteins plays a crucial role in opening up ligand-binding sites. This dynamic behavior is intimately linked to a variety of biological functions, including molecular docking, catalytic activity, and the transport of small molecules [59–64].

In a 200 ns MD simulation of p.Trp691Cys, we observed that the replacement of Trp691 with Cys691 generated new hydrogen bonds with adjacent residues. This increased the protein's structural stability but reduced its flexibility. Conversely, the p.Pro1431Argfs*8 mutation truncated the protein at the 1437th residue, resulting in fewer secondary structures and a reduced number of hydrogen bonds. Additionally, this variant altered the polarity of specific residues, such as changing the positively charged His1432 to a neutral Thr and replacing Glu1437 with a neutral Ser. These changes likely disrupted existing salt bridges, increasing the dynamism of the region between residues 1261 and 1385, thereby altering its spatial arrangement.

Our molecular docking study reveals the significant influence of H-bond interactions on the binding affinity between proteins and ligands. We found that four mutant proteins (p.Trp691Cys, p.Pro1431Argfs*8, p.Trp691Ala, and p.Pro1431Ala) have lower binding free energies and higher affinity for DKK1 compared to the wildtype. Specifically, the p.Trp691Cys and p.Trp691Ala variants show a markedly reduced binding free energy, emphasizing the importance of the Trp691 residue within the conserved third YWTD-EGF-β-propeller domain in the protein's function and structure. These findings suggest that these mutants could enhance their binding affinity and stability with DKK1, possibly playing a crucial role in modulating the Wnt signaling pathway [45]. The enhanced binding could lead to increased inhibition of LRP5 by DKK1, potentially suppressing Wnt signaling through the promotion of β-catenin degradation.

In our study, we observed that despite sharing the same pathogenic variant, probands and their parents exhibited different clinical phenotypes. Specifically, parents of patients with severe FEVR typically displayed mild manifestations, which is consistent with a previous report by Gilmour [2]. Additionally, probands from different families who harbored the same variant showed varying levels of severity in the disorder. For example, in our study, proband 4 carrying the *TSPAN12* variant (c.633 T > A: p.Tyr211*) experienced retinal detachment, while the same variant was detected by Zou et al. [51]. resulted in milder conditions such as vitreous hemorrhage and peripheral avascular area or vascular exudate. Furthermore, a separate study [50] reported that younger FEVR patients tend to display more severe phenotypes than older patients. Gilmour et al. [2] have speculated that individuals carrying the same pathogenic variant often present different phenotypes due to the influence of epigenetics and environmental factors. They found that even minor changes in oxygen content or intrauterine conditions, such as PaO2 alteration or exposure to certain drugs, may have a considerable impact on patients who harbor FEVR pathogenic variants.

Nevertheless, our study does have the following limitations that should be acknowledged. Firstly, the small sample size may potentially introduce sample selection bias and hinder the detection of rare pathogenic variants. Additionally, the limitations of the sequencing technology used may have hindered the detection of novel pathogenic variants or copy number variants related to FEVR. Furthermore, in vitro or animal laboratory testing is needed to confirm the pathogenicity of the two novel variants (c.2073G > T: p.Trp691Cys and c.4289delC: p.Pro1431Argfs*8) of *LRP5*. To address these limitations, future studies could expand the sample size and include a broader range of family members for TGS. Additionally, integrating laboratory testing would allow for a more comprehensive analysis of genetic variations.

## Conclusion

This study successfully identified two new pathogenic variants in the LRP5 gene (c.2073G > T: p.Trp691Cys and c.4289delC: p.Pro1431Argfs*8) associated with FEVR, leveraging both mutation screening and MD simulations. This expands our knowledge of FEVR-related mutations and opens new doors for genetic research in FEVR patients. We strongly advocate for family counseling and genetic education programs to offer comprehensive insights into the disease's genetic aspects for affected individuals and families. Periodic eye screenings are also recommended for early diagnosis and tailored treatment approaches. Emphasizing interdisciplinary collaboration can facilitate knowledge exchange and comprehensive patient care, aiming to enable early diagnosis, improve personalized treatments, and ultimately lower FEVR prevalence.

### Supplementary Information

**Additional file 1. **The trajectory animations depict the dynamic behavior of a wildtype protein and two mutant proteins (p.Trp691Cys and p.Pro1431Argfs8) during the molecular dynamics simulation.

**Additional file 2. **Visual Representation of DSSP Analysis During the course of a 200ns molecular dynamics simulation, discernible alterations were observed in the secondary structural elements of the wild-type, p.Trp691Cys and p.Pro1431Argfs*8 proteins. The diagram employs a color-coded scheme to facilitate easy interpretation of these secondary structures: White denotes 'coil'; Red indicates 'β-sheet'; Black signifies 'β-bridge'; Green marks 'bend'; Yellow highlights 'turn'; Blue stands for 'α-helix'; Purple designates '5-helix'; and Gray corresponds to '3-helix'.

**Additional file 3. **Comparisons of the hydrogen bond interactions and distance between wildtype-DKK1, p.Trp691Cys-DKK1, and p.Pro1431Argfs*8-DKK1 during molecular docking.

**Additional file 4. **Comparisons of the molecular docking results of the alanine mutagenesis study.

## Data Availability

The datasets generated and/or analyzed during the current study are available in the [National Library of Medicine] repository, (https://www.ncbi.nlm.nih.gov/sra/PRJNA922952).
