# Supplementary figures and images for: Genetic detection of two novel LRP5 pathogenic variants in patients with familial exudative vitreoretinopathy
Source: BMC Ophthalmol. 2023 Nov 29;23:489. doi: 10.1186/s12886-023-03243-2 (PMC10685552; doi:10.1186/s12886-023-03243-2)

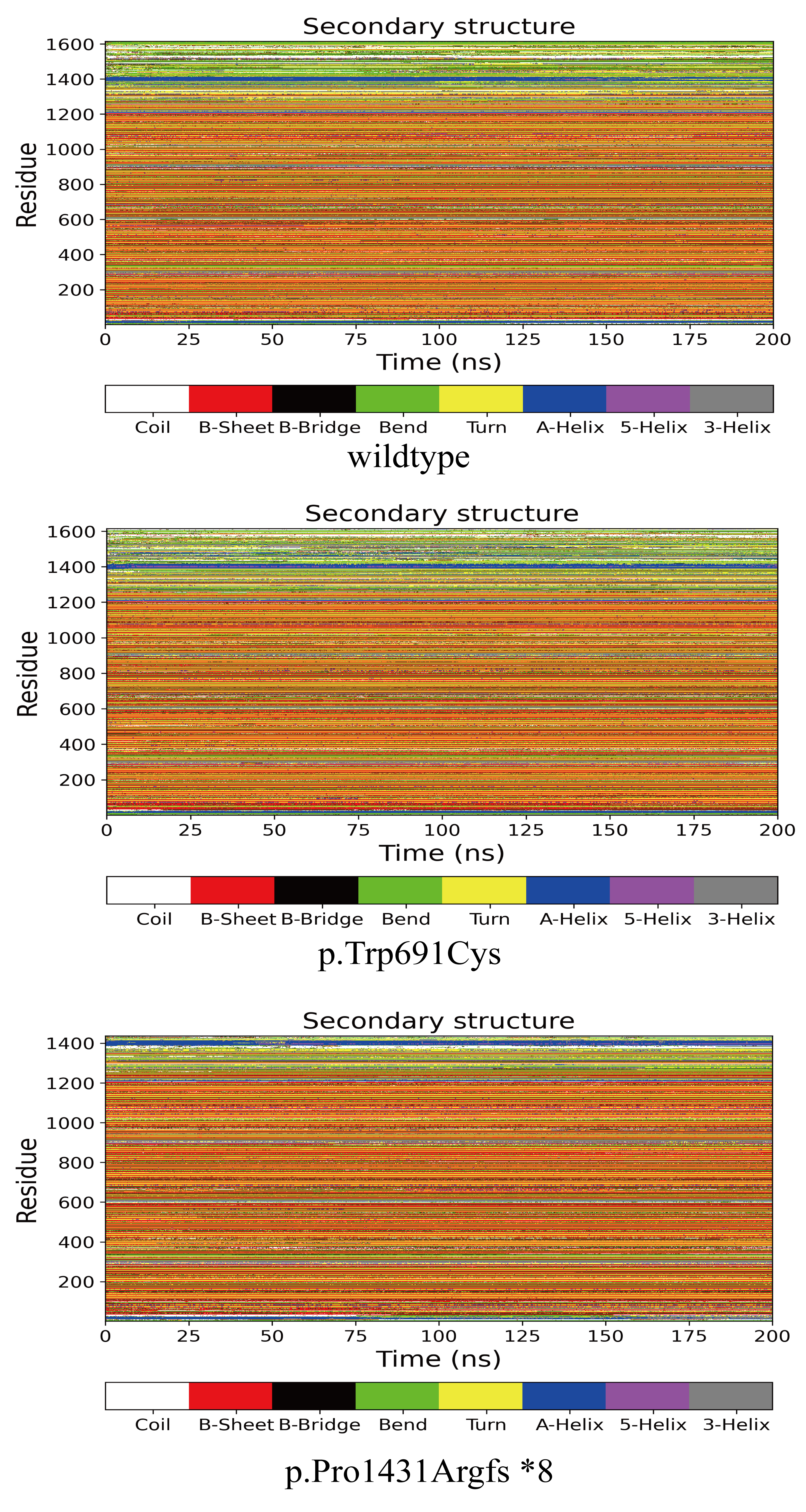

Supplement: Supplementary file 2 — Additional file 2. Visual Representation of DSSP Analysis During the course of a 200ns molecular dynamics simulation, discernible alterations were observed in the secondary structural elements of the wild-type, p.Trp691Cys and p.Pro1431Argfs*8 proteins. The diagram employs a color-coded scheme to facilitate easy interpretation of these secondary structures: White denotes 'coil'; Red indicates 'β-sheet'; Black signifies 'β-bridge'; Green marks 'bend'; Yellow highlights 'turn'; Blue stands for 'α-helix'; Purple designates '5-helix'; and Gray corresponds to '3-helix'. [file 12886_2023_3243_MOESM2_ESM.tif]
