# Supplementary material for: Genetic detection of two novel LRP5 pathogenic variants in patients with familial exudative vitreoretinopathy
Source: BMC Ophthalmol. 2023 Nov 29;23:489. doi: 10.1186/s12886-023-03243-2 (PMC10685552; doi:10.1186/s12886-023-03243-2)
Supplement: Supplementary file 3 — Additional file 3. Comparisons of the hydrogen bond interactions and distance between wildtype-DKK1, p.Trp691Cys-DKK1, and p.Pro1431Argfs*8-DKK1 during molecular docking. [file 12886_2023_3243_MOESM3_ESM.doc]

**Genetic detection of two novel LRP5 pathogenic variants in patients with familial exudative vitreoretinopathy**

Jiayu Li,1,2†, Chanjuan Wang,2†, Shaochi Zhang,2 Bo Cai,2 Bo Pan,2 Caihong Sun,1,2 Xiaolong Qi,2 Chunmei Ma,2 Wei Fang,2 Kangxin Jin,3 Xiaojun Bi,2* Zibing Jin,3* Wenjuan Zhuang1,2*

*1 Third Clinical Medical College of Ningxia Medical University, Shengli Street, Yinchuan, Ningxia, China; 2 Ningxia Eye Hospital, People’s Hospital of Ningxia Hui Autonomous Region, Huanghe Road, Yinchuan, 750011, Ningxia, China; 3 Beijing Institute of Ophthalmology, Beijing Tongren Eye Center, Beijing Tongren Hospital, Capital Medical University, Beijing, China.*

** Corresponding authors: Xiao-Jun Bi, bxj511@163.com; Zi-Bing Jin, jinzibing@foxmail.com; Wen-Juan Zhuang, zh_wenj@163.com.*

*† These authors contributed equally to this work.*

Additional File 3: Comparisons of the hydrogen bond interactions and distance between wildtype-DKK1, p.Trp691Cys-DKK1, and p.Pro1431Argfs*8-DKK1 during molecular docking.

| Wildtype-DKK1 | | p.Trp691Cys-DKK1 | | p.Pro1431Argfs*8-DKK1 | |
| --- | --- | --- | --- | --- | --- |
| H-bonds | distance(Å) | H-bonds | distance(Å) | H-bonds | distance(Å) |
| Glu829-Asn256 | 1.9 | Lys802-Gln253 | 2.4 | Lys 802-His252 | 2.0 |
| Gly782-Val219 | 1.9 | Lys802-Ser258 | 2.1 | Lys 802-Ser258 | 2.1 |
| Lys783-Gln248 | 2.0 | Asp822-Ser258 | 1.9 | Lys 802-Gln253 | 2.3 |
| Arg805-Arg236 | 1.8 | Gly782-Val219 | 2.0 | Glu829-Asn256 | 1.7 |
| Arg805-Phe234 | 2.0 | Trp780-Arg236 | 2.3 | Asp822-Arg259 | 1.7 |
| Asp824-Arg236 | 1.8 | Glu721-Arg236 | 2.6 | Asp824-Arg224 | 1.8 |
| Thr825-Arg225 | 1.9 | Ala677-Trp206 | 2.1 | Asn826-Arg259 | 2.7 |
| Asp843-Arg259 | 1.9 | Arg805-Glu232 | 1.9 | Trp780-Arg236 | 2.2 |
| Asp843-Arg225 | 1.9 | Tyr719-His204 | 1.8 | Gly781-Arg236 | 1.8 |
| Leu845-Arg225 | 1.8 | Tyr719-Trp206 | 2.4 | Lys783-Gly217 | 1.8 |
| Pro846-Arg225 | 2.2 | Gln1175-Gln184 | 2.0 | Glu721-His204 | 1.8 |
| Asn864-Lys226 | 1.9 | Lys953-Thr181 | 1.9 | Trp863-Leu231 | 2.4 |
|  |  | Arg1194-Glu185 | 1.9 |  |  |
