# Supplementary material for: Genetic detection of two novel LRP5 pathogenic variants in patients with familial exudative vitreoretinopathy
Source: BMC Ophthalmol. 2023 Nov 29;23:489. doi: 10.1186/s12886-023-03243-2 (PMC10685552; doi:10.1186/s12886-023-03243-2)
Supplement: Supplementary file 4 — Additional file 4. Comparisons of the molecular docking results of the alanine mutagenesis study. [file 12886_2023_3243_MOESM4_ESM.docx]

**Genetic detection of two novel LRP5 pathogenic variants in patients with familial exudative vitreoretinopathy**

Jiayu Li,1,2†, Chanjuan Wang,2†, Shaochi Zhang,2 Bo Cai,2 Bo Pan,2 Caihong Sun,1,2 Xiaolong Qi,2 Chunmei Ma,2 Wei Fang,2 Kangxin Jin,3 Xiaojun Bi,2* Zibing Jin,3* Wenjuan Zhuang1,2*

*1 Third Clinical Medical College of Ningxia Medical University, Shengli Street, Yinchuan, Ningxia, China; 2 Ningxia Eye Hospital, People’s Hospital of Ningxia Hui Autonomous Region, Huanghe Road, Yinchuan, 750011, Ningxia, China; 3 Beijing Institute of Ophthalmology, Beijing Tongren Eye Center, Beijing Tongren Hospital, Capital Medical University, Beijing, China.*

** Corresponding authors: Xiao-Jun Bi, bxj511@163.com; Zi-Bing Jin, jinzibing@foxmail.com; Wen-Juan Zhuang, zh_wenj@163.com.*

*† These authors contributed equally to this work.*

Additional File 4: Comparisons of the molecular docking results of the alanine mutagenesis study.

| System name | wildtype-DKK1 | p.Trp691Ala-DKK1 | p.Pro1431Ala-DKK1 |
| --- | --- | --- | --- |
| ΔEvdw | -161.88 ±4.45 | -239.39±3.85 | -178.89±4.66 |
| ΔEelec | -3151.37±21.50 | -3931.55±15.49 | -3566.94±20.35 |
| ΔGGB | 3245.26±21.90 | 4071.77±15.40 | 3674.66±20.99 |
| ΔGSA | -22.37±0.69 | -35.57±0.60 | -24.37±0.61 |
| ΔGbind | -90.37±2.35 | -134.75±3.96 | -95.54±3.18 |

ΔEvdW: van der Waals energy.

ΔEelec: electrostatic energy.

ΔGGB: electrostatic contribution to solvation.

ΔGSA: non-polar contribution to solvation.

ΔGbind: binding free energy.
